# Supplementary material for: Changes in the Vaginal Microbiome During Pregnancy and the Postpartum Period in South African Women: a Longitudinal Study
Source: Reprod Sci. 2023 Sep 18;31(1):275–87. doi: 10.1007/s43032-023-01351-4 (PMC10784382; doi:10.1007/s43032-023-01351-4)
Supplement: Supplementary file 1 — (DOCX 1408 kb) [file 43032_2023_1351_MOESM1_ESM.docx]

**Changes in the vaginal microbiome during pregnancy and the postpartum period in South African women: a longitudinal study**

Katherine T Li^1^, Fan Li^2^, Heather Jaspan^3,4,5,6^, Dorothy Nyemba^7^, Landon Myer^6,7^, Grace Aldrovandi^2^, and Dvora Joseph-Davey^1,7,8^*

1 Division of Infectious Disease, David Geffen School of Medicine, University of California Los Angeles, Los Angeles, California, USA

2 Department of Pediatric Infectious Diseases, David Geffen School of Medicine, University of California Los Angeles, Los Angeles, California, USA

3 Department of Pathology, Institute of Infectious Disease and Molecular Medicine, University of Cape Town, South Africa

4 Departments of Pediatrics and Global Health, University of Washington, Seattle, Washington, USA

5 Center for Global Infectious Disease Research, Seattle Children’s Research Institute, Seattle, Washington, USA

6 Institute of Infectious Disease and Molecular Medicine, University of Cape Town, South Africa

7 Division of Epidemiology and Biostatistics, School of Public Health, University of Cape Town, Cape Town, South Africa

8 Department of Epidemiology, Fielding School of Public Health, University of California Los Angeles, USA

*Corresponding author

**SUPPLEMENTAL MATERIAL**

Table of Contents

[Supplement S1: Distribution of gestational age at Visit A (n = 242) 3](#_Toc141783463)

[Supplement S2: STI diagnoses by visit (n = 242) 4](#_Toc141783464)

[Supplement S3: Principal Coordinates Analysis by CST (n = 687 samples) 5](#_Toc141783465)

[Supplement S4: CST distribution by gestational age at Visit A (initial antenatal visit, n = 242) 6](#_Toc141783466)

[Supplement S5: Stability and transition probability of CSTs, stratified by HIV status and STI diagnosis 7](#_Toc141783467)

[Supplement S6: Differential abundance testing of microbial species in women with and without HIV (zero-inflated negative binomial model) 8](#_Toc141783468)

[Supplement S7: microbiome profiles with respect to ART status at Visit A 9](#_Toc141783469)

[Supplement S8: CST distribution of six women who seroconverted to HIV positive by Visit PPt 10](#_Toc141783470)

[Supplement S9: CST distribution with respect to diagnosis of individual STI at any visit 11](#_Toc141783471)

[Supplement S10: Differential abundance testing of vaginal microbial species in women with and without STI diagnosis (zero-inflated negative binomial model) for: 11](#_Toc141783472)

[Supplement S11: Microbiome profiles by antibiotics received at the prior visit 14](#_Toc141783473)

[Supplement S12: Microbiome profiles stratified by adverse birth outcome 15](#_Toc141783474)

# Supplement S1: Distribution of gestational age at Visit A (n = 242)


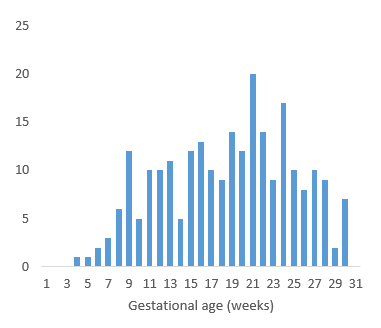


# Supplement S2: STI diagnoses by visit (n = 242)

|  | Visit A | % | Visit B | % | Visit PPt | % |
| --- | --- | --- | --- | --- | --- | --- |
| C trachomatis | 49 | 84% | 5 | 9% | 4 | 7% |
| N gonorrhea | 14 | 70% | 3 | 15% | 3 | 15% |
| T vaginalis | 37 | 82% | 6 | 13% | 2 | 4% |
| Total* | 100 | 81% | 14 | 11% | 9 | 7% |

*Total STIs diagnosed at each visit includes co-infections in the same participant

# Supplement S3: Principal Coordinates Analysis by CST (n = 687 samples)


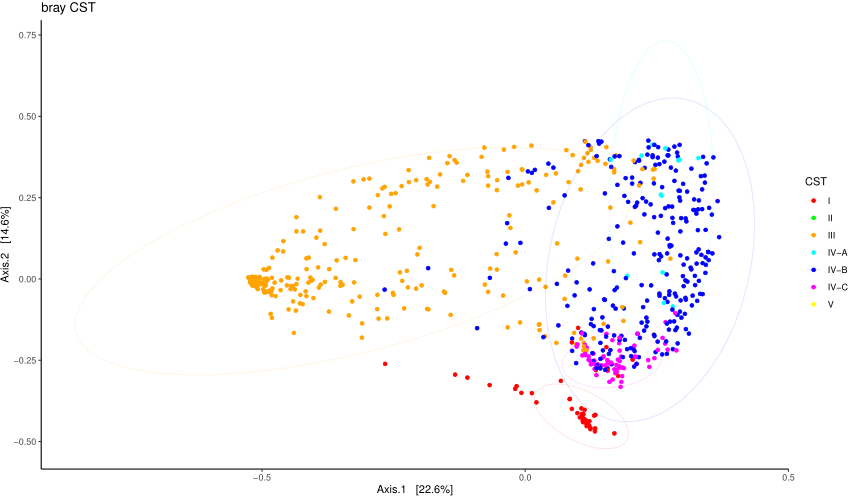


# Supplement S4: CST distribution by gestational age at Visit A (initial antenatal visit, n = 242)


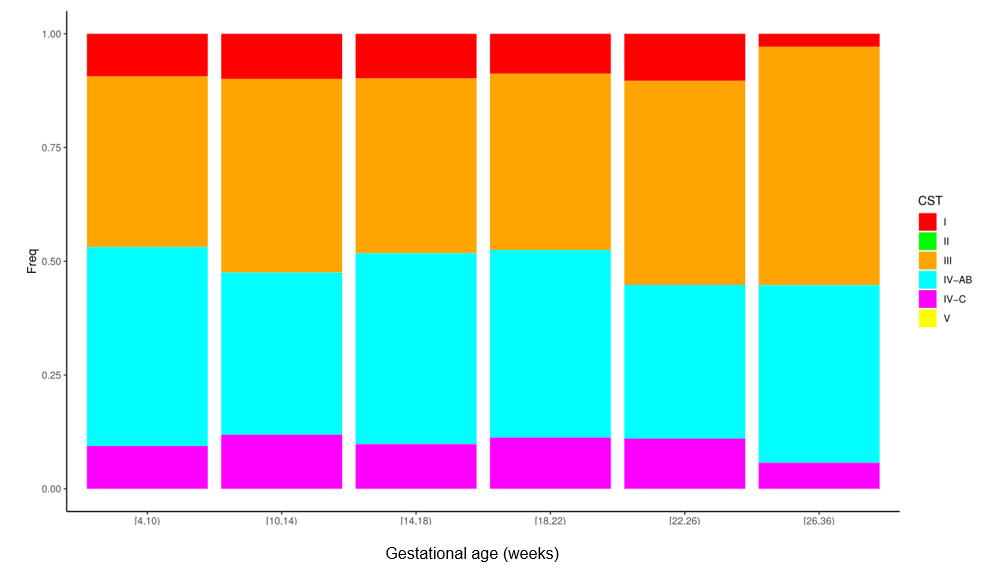


# Supplement S5: Stability and transition probability of CSTs, stratified by HIV status and STI diagnosis

1. **Stability of CSTs from Visit A to Visit B, and Visit B to Visit PPt**

| CST | Transition | Observed stability | Expected stability | Adjusted p-value |
| --- | --- | --- | --- | --- |
| I | A -> B | 15 | 10.12 | 0.040 |
| III | A -> B | 75 | 61.84 | 0.001 |
| IV-AB | A -> B | 32 | 48.35 | 3.70E-05 |
| IV-C | A -> B | 0 | 1.69 | 0.188 |
| I | B -> PPt | 1 | 8.38 | 0.006 |
| III | B -> PPt | 23 | 30.56 | 0.035 |
| IV-AB | B -> PPt | 28 | 12.82 | 4.18E-07 |
| IV-C | B -> PPt | 0 | 0.25 | 1.000 |

1. **Impact of HIV status, STI diagnosis, and Adverse birth outcome on transitions between CSTs and Stability across visits**

| Transition | Variable | P-adjusted |
| --- | --- | --- |
| CST IV at Visit A to CST I or III at Visit B | HIV status | 0.931 |
|  | STI diagnosis | 0.472 |
|  | Adverse outcome | 0.964 |
| CST I or III at Visit B to CST IVC at Visit PPt | HIV status | 0.422 |
|  | STI diagnosis | 0.422 |
|  | Adverse outcome | 0.444 |
| Stability across Visits A, B, and PPt | HIV status | 0.525 |
|  | STI diagnosis | 0.864 |
|  | Adverse outcome | 0.931 |

# Supplement S6: Differential abundance testing of microbial species in women with and without HIV (zero-inflated negative binomial model)


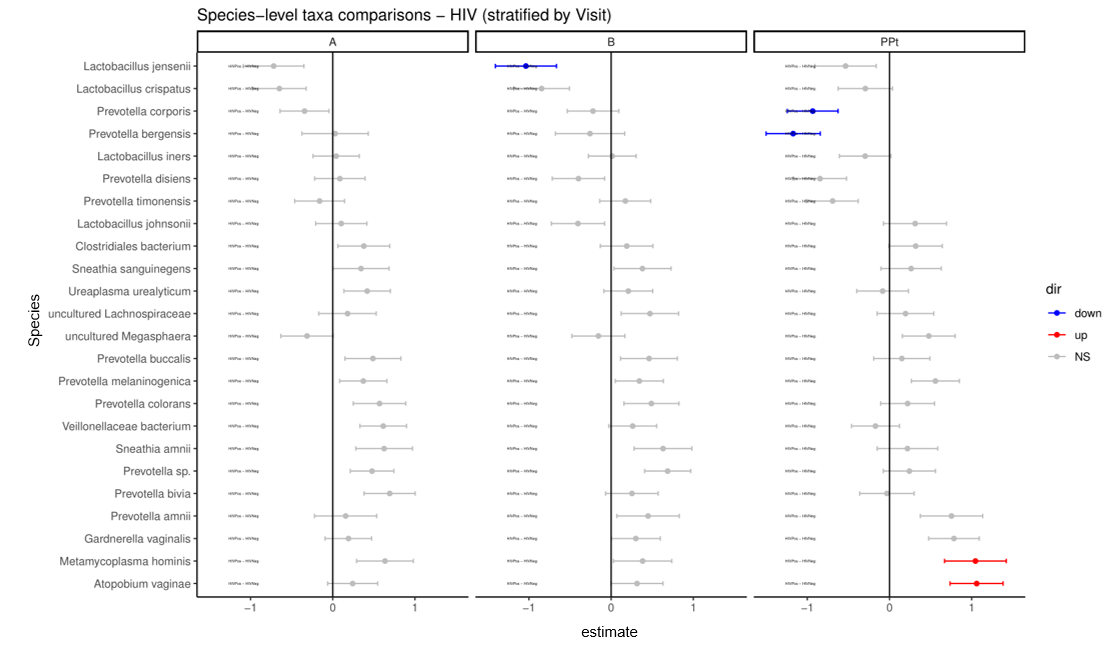


# Supplement S7: microbiome profiles with respect to ART status at Visit A

1. **CSTs at Visit A with respect to ART status**


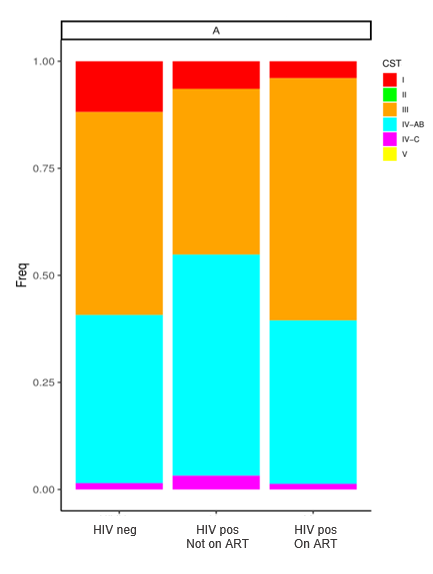


1. **Shannon diversity with respect to ART status**


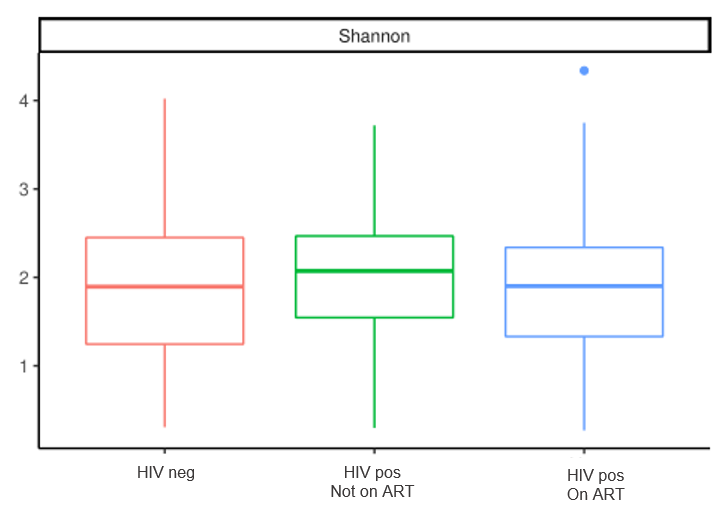


# Supplement S8: CST distribution of six women who seroconverted to HIV positive by Visit PPt


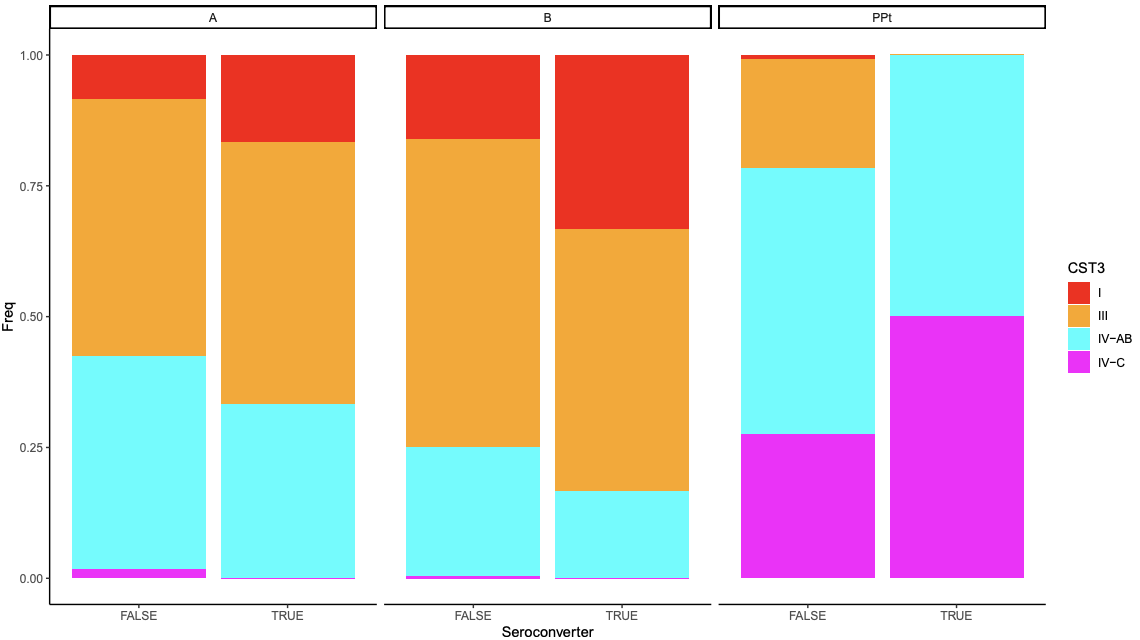


# Supplement S9: CST distribution with respect to diagnosis of individual STI at any visit

1. **Chlamydia trachomatis (CT)**


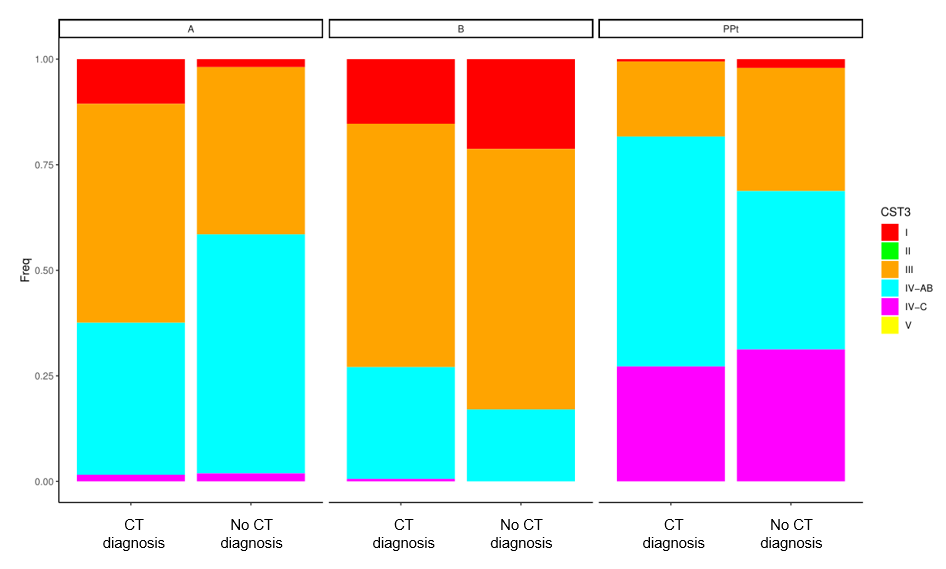


1. **Neisseria gonorrhea (NG)**


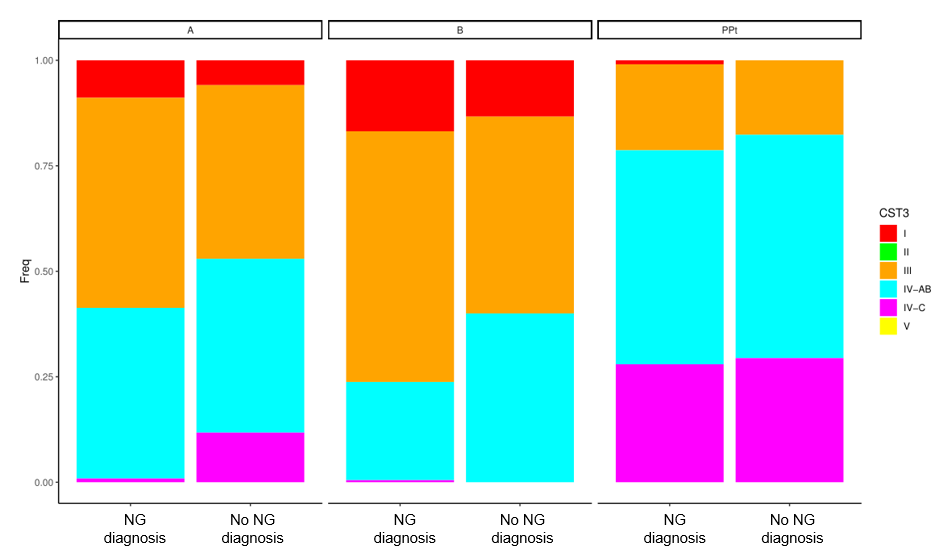


1. **Trichomonas vaginalis (TV)**

#
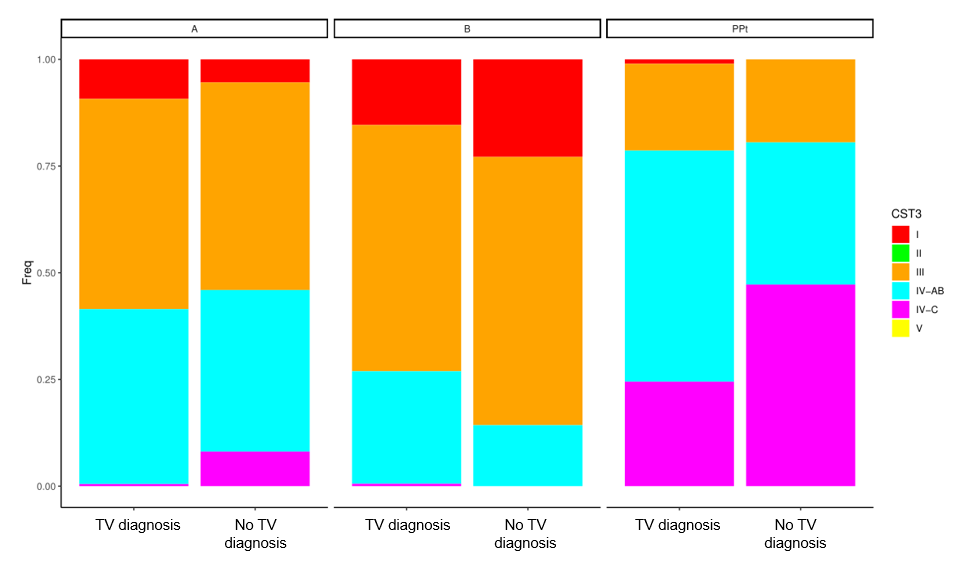
Supplement S10: Differential abundance testing of vaginal microbial species in women with and without STI diagnosis (zero-inflated negative binomial model) for:

1. **Any STI**


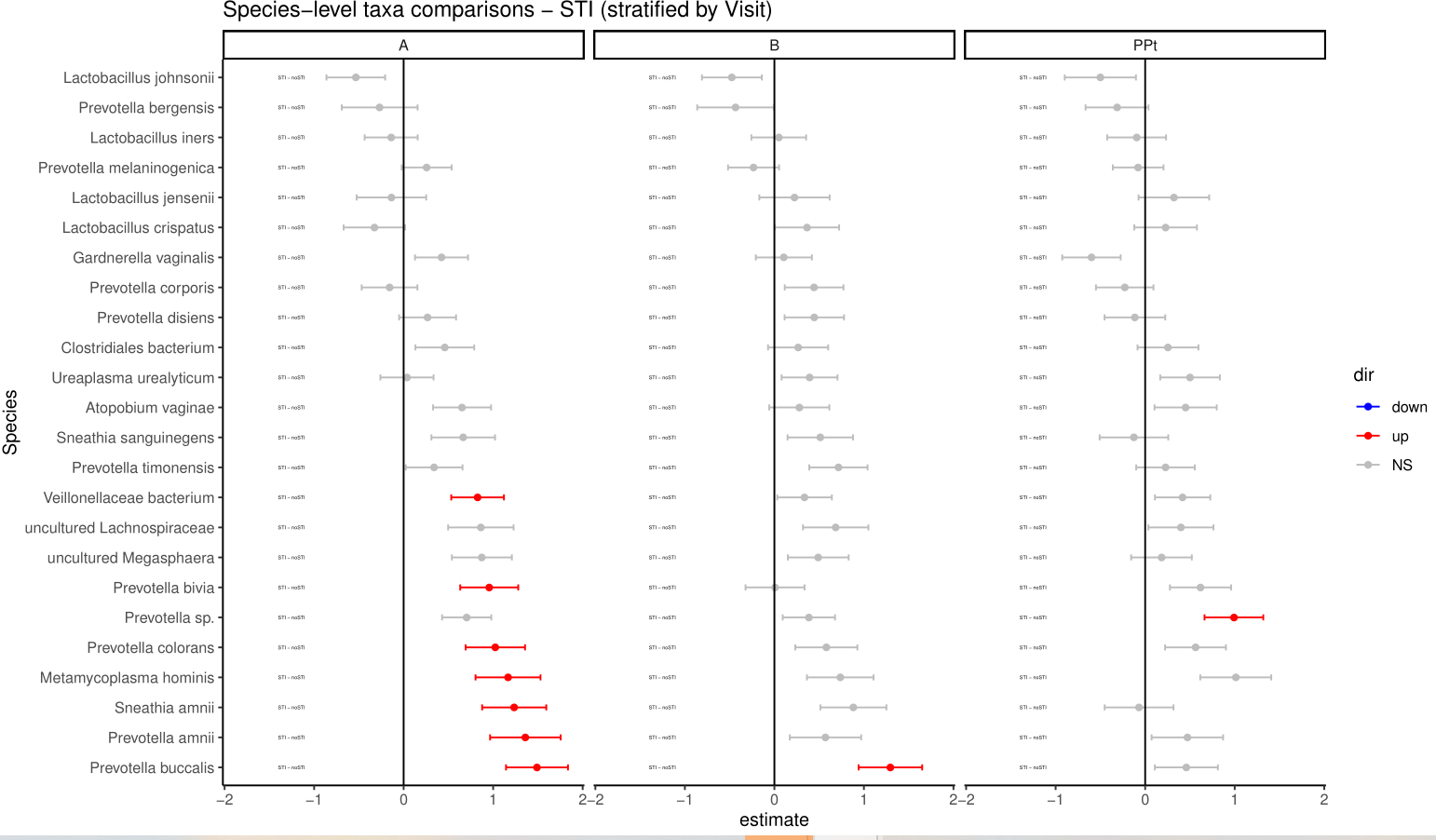


1. **Chlamydia trachomatis (CT) infection**


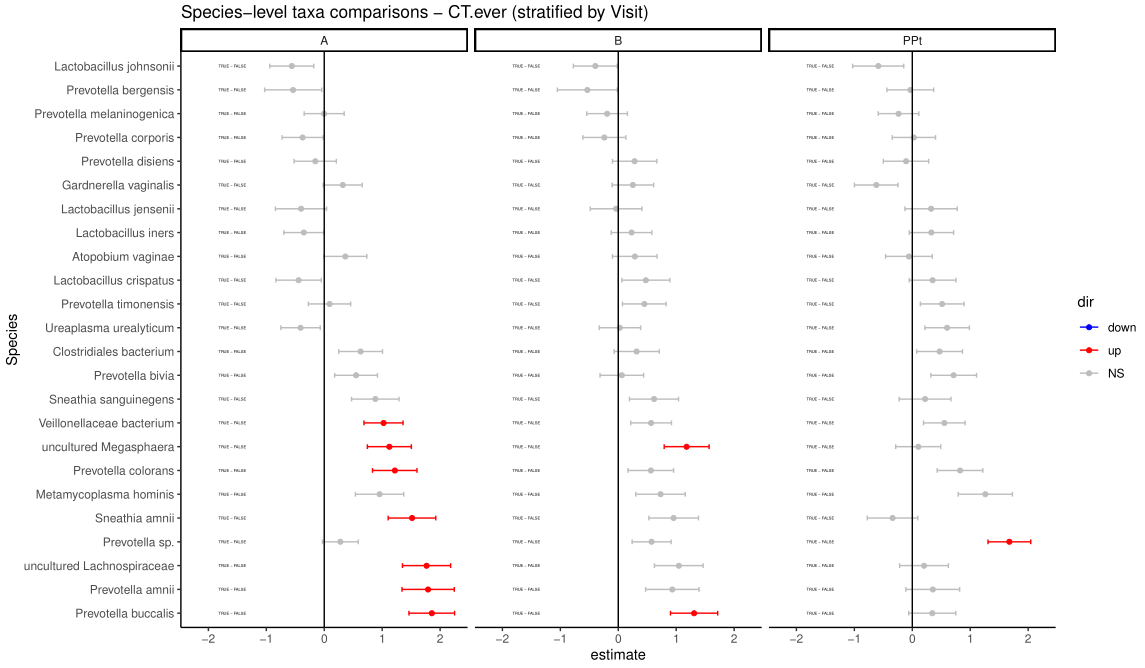


1. **Neisseria gonorrhea (NG) infection**


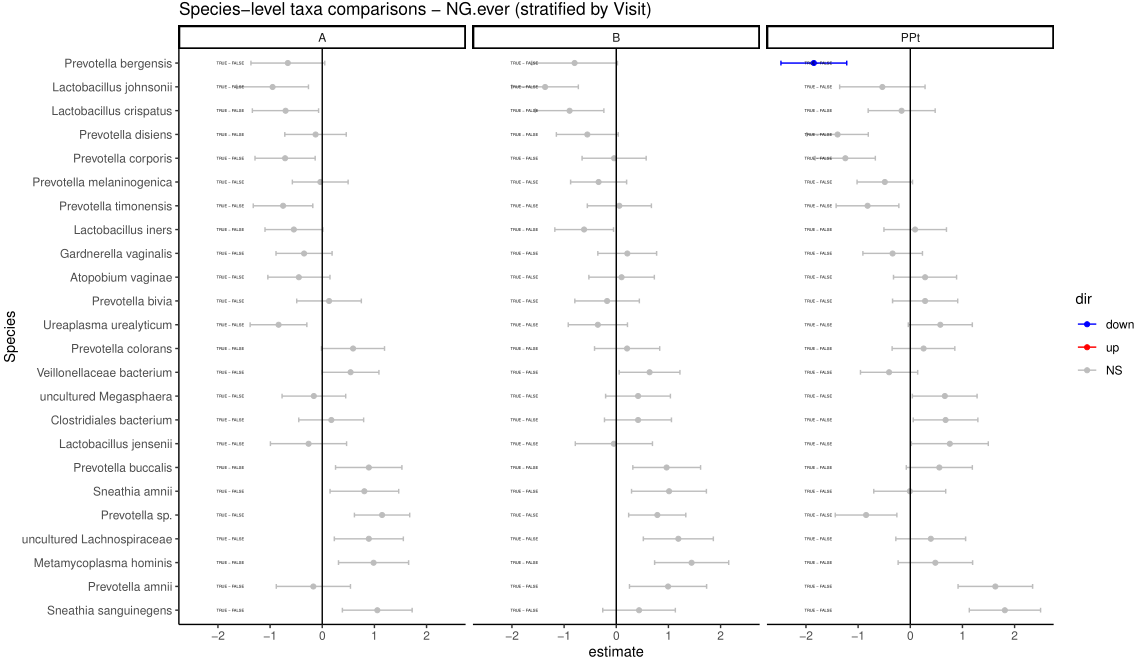


1. **Trichomonas vaginalis (TV) infection**


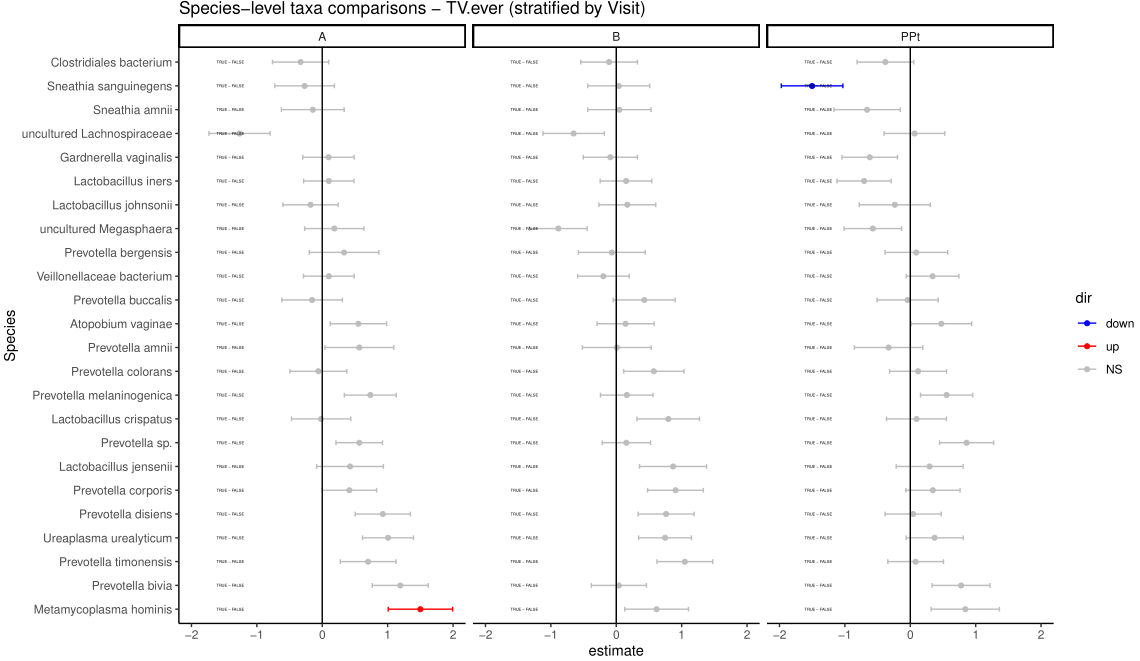


# Supplement S11: Microbiome profiles by antibiotics received at the prior visit

59 women reported possible STI-related symptoms at Visit A, of which 17 had prior treatment. The rest received empiric syndromic management as part of the study, and of those, 17 ultimately did not test positive for any STI. At Visit B, there were 14 women who reported STI symptoms, of which 8 were previously treated, and only 1 woman who was empirically treated as part of the study did not test positive for any STI.

1. **CST distribution by antibiotics received at prior visit**


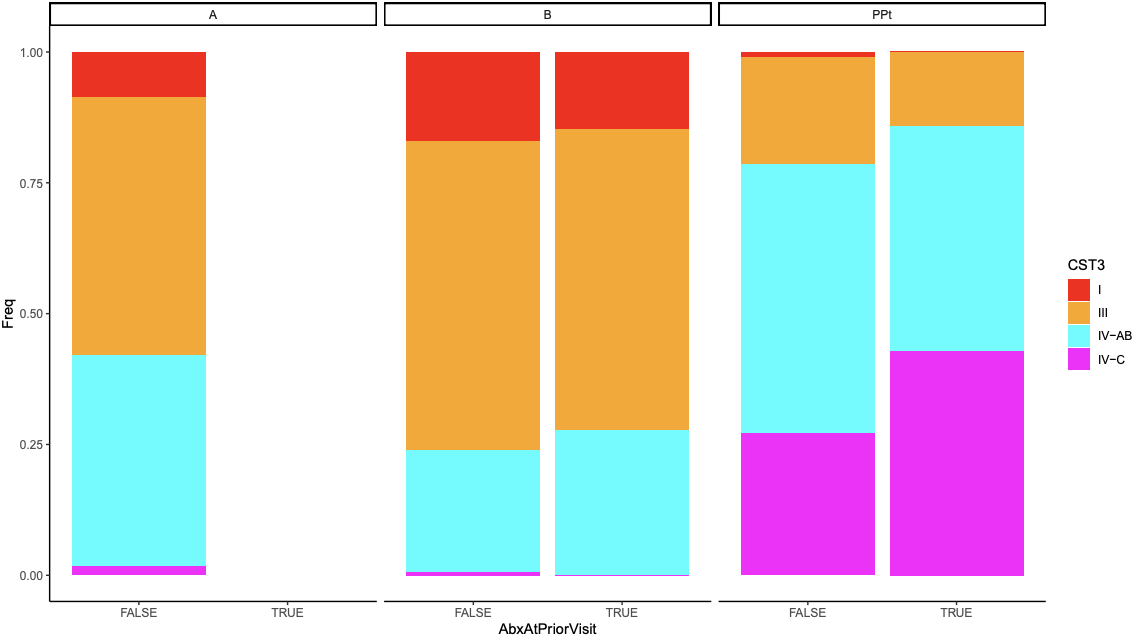


1. **Principal coordinates analysis by antibiotics received at prior visit**

R2 = 0.00038

p = 0.871


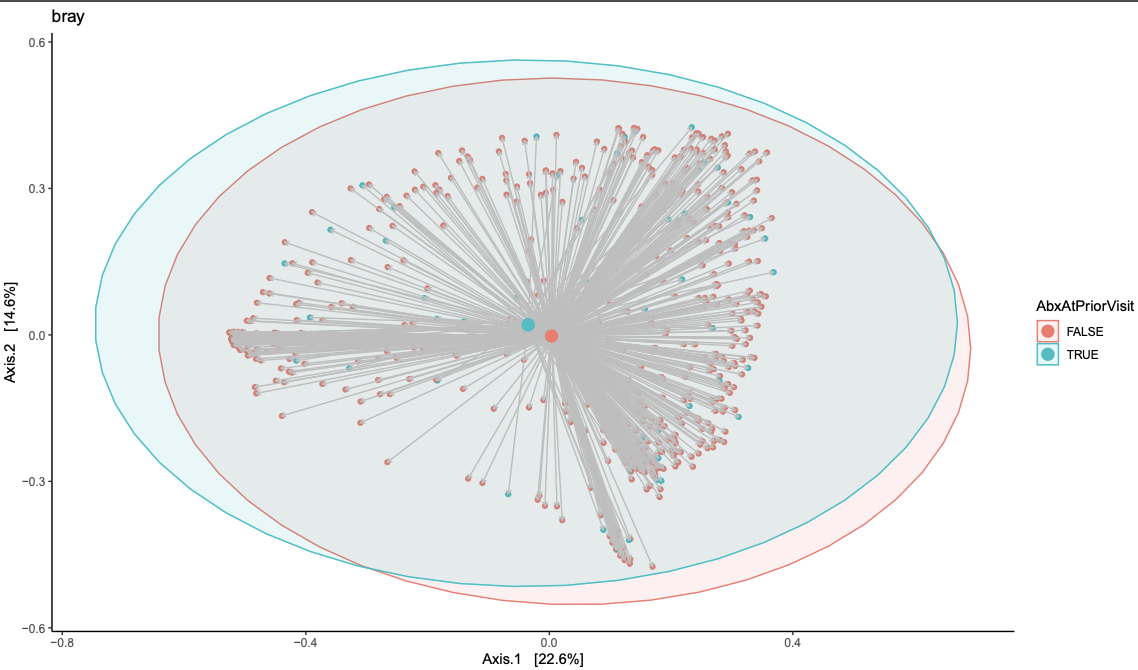


1. **Shannon diversity by antibiotics received at prior visit**


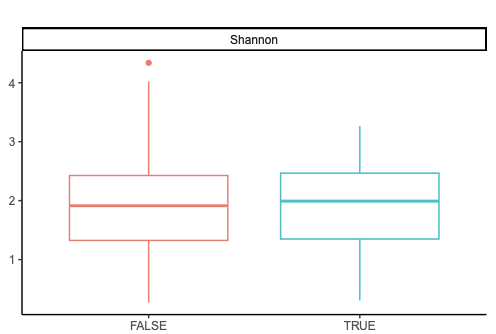

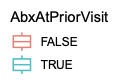


#
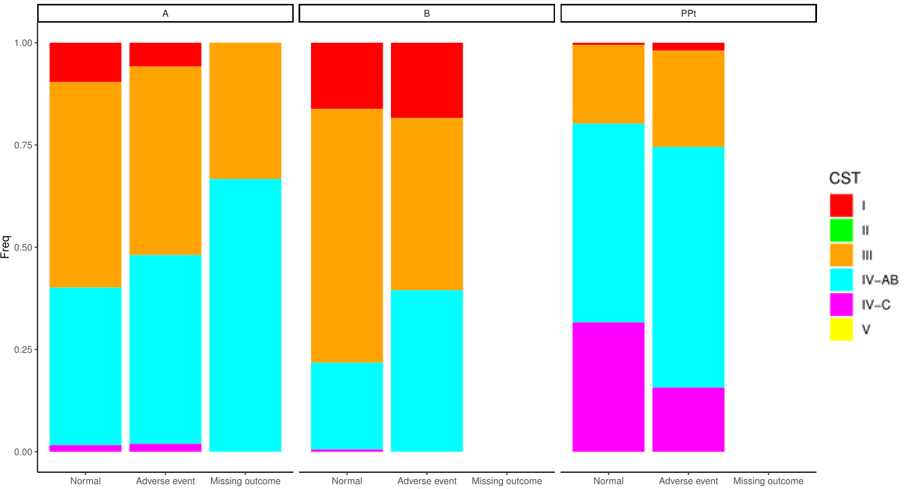
Supplement S12: Microbiome profiles stratified by adverse birth outcome

**a) CST distribution by adverse outcomes, by visit**


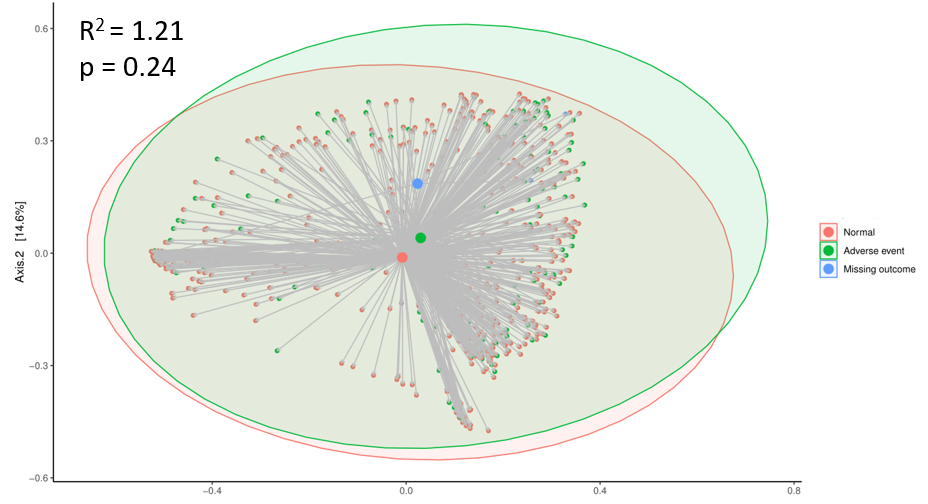

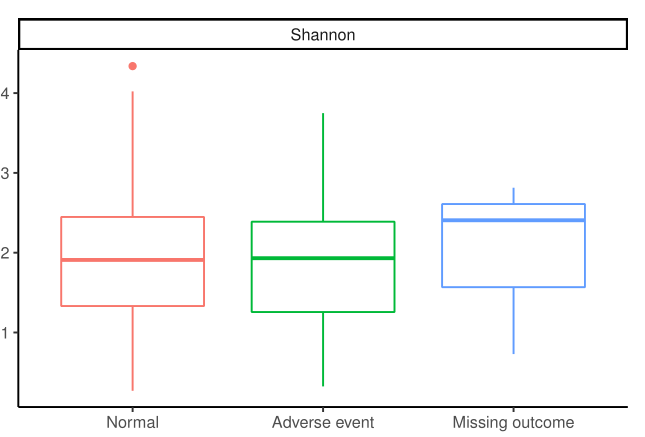

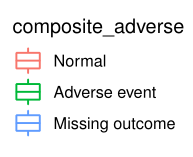


**c) Shannon diversity by adverse outcome**

**b) Principal coordinates analysis by adverse outcome**
